
JOURNAL INFORMATION
==============================
NLM Title Abbreviation: Acta Crystallogr C Struct Chem
Journal ID: Acta Crystallogr C Struct Chem
NLM Journal ID: 101620313
Journal ID: Acta Cryst. C

Acta Crystallographica Section C: Structural Chemistry

EISSN: 2053-2296
Publisher: International Union of Crystallography

ARTICLE INFORMATION
==============================
PMCID: PMC12138255
PMID: 40434738
DOI: 10.1107/S2053229625004309
Article ID: fp3097
Article ID: S2053229625004309
Article version: 1
Subjects: Research Papers

Synthesis and structure analysis of a cobalt(III) coordination com­pound obtained from a redox-active phenolate ligand and cobalt(II)

Cobalt(III) compound from a redox-active ligand and cobalt(II)

Bruzeguini Carlos Eduardo Tartaglia a
Bezerra Rodrigo Iraha a
Ribeiro Marcos Antonio a *
a Universidade Federal do Espírito Santo , Av. Fernando Ferrari, 514 Goiabeiras, Vitória, Esprito Santo, 29075190, Brazil
Almeida Paz F. A. Editor
University of Aveiro, Portugal
Correspondence e-mail: marcos.a.ribeiro@ufes.br
Publication date: 2025 Jun 1
Electronic publication date: 2025 May 28
Volume: 81
Issue: Pt 6
First page: 358
Last page: 364
Received 2024 Sep 12; Accepted 2025 May 13
Copyright: © Bruzeguini et al. 2025
Copyright year: 2025
License: This is an open-access article distributed under the terms of the Creative Commons Attribution (CC-BY) Licence, which permits unrestricted use, distribution, and reproduction in any medium, provided the original authors and source are cited.
License URL: https://creativecommons.org/licenses/by/4.0/

A full version of this article is available from Crystallography Journals Online.
Page count: 7

==============================

Acta Cryst C STRUCTURAL CHEMISTRY

ISSN 2053-2296

### Synthesis and structure analysis of a cobalt(III) coordination compound obtained from a redox-active phenolate ligand and cobalt(II)

**Carlos Eduardo Tartaglia Bruzeguini, Rodrigo Iraha Bezerra and Marcos Antonio Ribeiro***

Universidade Federal do Espírito Santo, Av. Fernando Ferrari, 514 Goiabeiras, Vitória, Esprito Santo, 29075190, Brazil. *Correspondence e-mail: marcos.a.ribeiro@ufes.br

Received 12 September 2024
Accepted 13 May 2025

Edited by F. A. Almeida Paz, University of Aveiro, Portugal

**Keywords:** crystal structure; redox-active ligand; cobalt; coordination compound; valence tautomerism.

**CCDC reference:** 2382866

**Supporting information:** this article has supporting information at journals.iucr.org/c

In an attempt to synthesize the coordination compound [Co($H_4L$)$_2$] using Co($ClO_4$)$_2$·6$H_2O$ and 2,4-di-*tert*-butyl-6-({2-[(3,5-di-*tert*-butyl-2-hydroxyphenyl)-amino]ethyl}amino)phenol ($H_4L$), we instead obtained the compound bis{2-[(2-aminoethyl)amino]-4,6-di-*tert*-butylphenolato-$\kappa^3N,N',O$}cobalt(III) perchlorate, [Co($C_{16}H_{27}N_2O$)$_2$]$ClO_4$. The compound was formed through the reaction of $Co^{II}$ with the decomposition product of $H_4L$. Using the structural data, we analyzed the behaviour of the redox-active ligand and the inter- and intramolecular interactions.

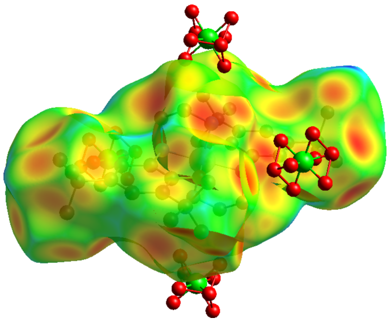

OPEN ACCESS

Published under a CC BY 4.0 licence

### 1. Introduction

Redox-active ligands are species capable of reversibly reacting, accepting and donating electrons. Due to this characteristic, redox-active ligands can sometimes act as electron sources. The scientific interest in this class of ligands arises from their capacity to access various electronic states. Examples of redox-active ligands are derivatives of catechol, naphthoquinones and aminophenol (Broere *et al.*, 2015; Nasibipour *et al.*, 2021).

Coordination compounds containing redox-active ligands and transition metals can form species with poorly defined oxidation states. This ambiguity often results from the small difference in the energies of valence orbitals of the ligands and metals. Consequently, coordination compounds formed by a transition metal and *o*-aminophenol derivatives can exhibit electronic structures with properties suitable for applications such as reaction catalysts, molecular magnets and models to mimic enzymatic reactions (Shultz, 2003; Pour *et al.*, 2020; Tezgerevska *et al.*, 2019; Gransbury *et al.*, 2019).

Given these properties, there is significant interest in exploring the electronic flexibility of compounds formed by cobalt and redox-active ligands, as this composition can show a valence tautomerism (VT) transition. VT transitions are characterized by a reversible intramolecular electron transfer between the metal ion and the redox-active ligand, accompanied by changes in the multiplicity of the compound. This phenomenon has attracted considerable attention recently due to its potential application in information storage devices (Panja *et al.*, 2016; Hathwar *et al.*, 2017). Examples of VT compounds of cobalt and *o*-aminophenol derivatives can be found in the literature, where multidentate ligands have been used as redox-active ligands.

Herein, we report the formation of a coordination compound between cobalt and the ligand 2-[(2-aminoethyl)amino]-4,6-di*tert*-butylphenol (H*L*), which is a degradation product of 2,4-di-*tert*-butyl-6-({2-[(3,5-di-*tert*-butyl-2-hydroxyphenyl)amino]ethyl}amino)phenol ($H_4L$). The results obtained from single-crystal X-ray diffraction for bis{2-[(2-aminoethyl)amino]-4,6-di-*tert*-butylphenolato-$\kappa^3N,N',O$}cobalt(III) perchlorate, $[Co(L)_2]ClO_4$, **I** (Scheme 1), enabled us to propose an explanation for the degradation reaction and the formation of the final compound  ion (see Fig. S2 in the supporting information).

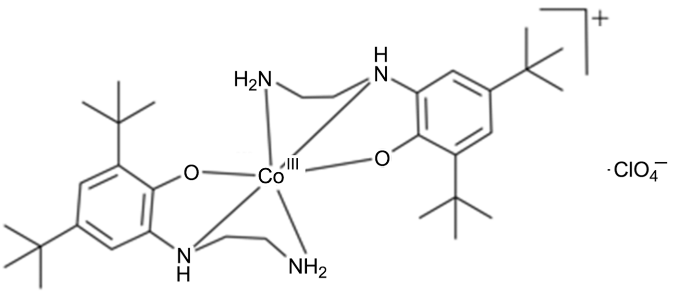

**I**

Scheme 1

### 2. Experimental

All reactants and solvents were used as received without further purification. The vibration spectra (FT–IR) were recorded in the 4000–400 $cm^{-1}$ region with a resolution of 2 $cm^{-1}$ and 64 scans using an Agilent Cary 630 spectrometer. The Hirshfeld surfaces were calculated using *CrystalExplorer* (Version 21.5; Spackman & Jayatilaka, 2009; Spackman *et al.*, 2021).

### 2.1. Synthesis and crystallization

The $H_4L$ ligand was prepared following the method described by Min *et al.* (2004). In a 50 ml round-bottomed flask, 3,5-di-*tert*-butylcatechol (6.750 mmol) was dissolved in acetonitrile (MeCN, 10 ml), resulting in a green solution. A solution of ethylenediamine (3.375 mmol) in MeCN (1 ml) was then added slowly. The resulting solution turned blue and was kept under reflux (373 K) and stirred for 1 h. After this, heating was stopped and the mixture was stirred at room temperature for an additional 3 h. A white solid formed during the reaction. This solid was filtered off under reduced pressure, washed with cold *n*-hexane and dried in an oven at 60 °C for 12 h. The material was weighed and a yield of 60% was calculated. FT–IR (ATR, $cm^{-1}$): 3478 $\nu$(O—H), 3344 $\nu$(N—H), 2958 $\nu$(C—H), 2927 $\nu$(C—H), 2869 $\nu$(C—H), 1591 $\nu$(C—O), 1418 $\nu$(N—H). $^1$H NMR (400 MHz, $CdCl_3$): $\delta$ 7.26 (*s*, $CdCl_3$), 7.05 (*s*, 1H), 7.04 (*s*, 1H), 6.88 (*s*, 1H), 6.88 (*s*, 1H), 3.33 (*s*, 1H), 1.58 (*s*, 1H), 1.40 (*s*, 1H), 1.23 (*s*, 1H). Solubility: methanol, ethanol, acetonitrile and toluene. Low solubility: dichloromethane and acetone.

In a round-bottomed flask, $H_4L$ (0.25 mmol) was dissolved in methanol (5 ml), resulting in a green solution. Subsequently, $Co(ClO_4)_2{\cdot}6H_2O$ (0.25 mmol) was added, causing the solution to change colour to dark red. An additional 5 ml of methanol was then added. The reaction mixture was heated at 50 °C for 2 h. No formation of precipitate was observed during this period. The solution was microfiltered and stored in a test tube at 15 °C for crystallization. Single crystals suitable for single-crystal X-ray diffraction were harvested after 14 days.

**Table 1**
Experimental details.

| | |
|---|---|
| Crystal data | |
| Chemical formula | $[Co(C_{16}H_{27}N_2O)_2]ClO_4$ |
| $M_r$ | 685.17 |
| Crystal system, space group | Monoclinic, $P2_1/c$ |
| Temperature (K) | 250 |
| *a*, *b*, *c* (Å) | 15.4774 (10), 10.4931 (4), 11.5251 (5) |
| $\beta$ (°) | 108.170 (6) |
| *V* (Å$^3$) | 1778.41 (16) |
| *Z* | 2 |
| Radiation type | Cu $K\alpha$ |
| $\mu$ ($mm^{-1}$) | 4.84 |
| Crystal size (mm) | 0.09 × 0.08 × 0.03 |
| Data collection | |
| Diffractometer | Rigaku XtaLAB Synergy Dualflex diffractometer with a HyPix detector |
| Absorption correction | Analytical [*CrysAlis PRO* (Rigaku OD, 2022) based on expressions derived by Clark & Reid (1995)] |
| $T_{min}$, $T_{max}$ | 0.953, 0.983 |
| No. of measured, independent and observed [$I > 2\sigma(I)$] reflections | 18781, 3366, 2856 |
| $R_{int}$ | 0.076 |
| $(\sin \theta/\lambda)_{max}$ (Å$^{-1}$) | 0.610 |
| Refinement | |
| $R[F^2 > 2\sigma(F^2)]$, $wR(F^2)$, *S* | 0.067, 0.173, 1.11 |
| No. of reflections | 3366 |
| No. of parameters | 234 |
| No. of restraints | 77 |
| H-atom treatment | H atoms treated by a mixture of independent and constrained refinement |
| $\Delta\rho_{max}$, $\Delta\rho_{min}$ (e Å$^{-3}$) | 0.62, −0.47 |

Computer programs: *CrysAlis PRO* (Rigaku OD, 2022), *SHELXT2018* (Sheldrick, 2015*a*), *SHELXL2018* (Sheldrick, 2015*b*), *OLEX2* (Dolomanov *et al.*, 2009), *Mercury* (Macrae *et al.*, 2020) and *OLEX2* (Dolomanov *et al.*, 2009).

### 2.2. Refinement

Crystal data, data collection and structure refinement details are summarized in Table 1. H atoms were placed in idealized positions and refined using a riding model, except for the amine H atoms, which were refined freely with a restrained N—H distance of 0.8 Å. The chloride ion exhibited significant disorder due to its position on an inversion centre. Each atom in the disordered component was assigned an occupancy of 0.5. Chemically equivalent bond lengths and angles were constrained to identical values and bond distance restraints were also applied. Additionally, the atomic displacement parameters for the disordered atoms were refined using a restraint.

### 2.3. $^1$H NMR data

The NMR spectra were recorded on a Varian 400 MHz spectrometer equipped with a 5 mm Broadband probe and dedicated channels for $^1$H. The sample was prepared by dissolving 9 mg of $H_4L$ in 700 µl of deuterated trichloromethane ($CDCl_3$; Sigma–Aldrich). Chemical shifts were reported in parts per million (ppm) relative to signals from residual hydrogen nuclei from the solvent and referenced to tetramethylsilane (TMS). The *MestreNova* software (Version 6.0; Mestreb Research S.L.U.; https://mestrelab.com/main-product/mnova) was used to process the data.

### 3. Results and discussion

### 3.1. Characterization of $H_4L$

The $^1$H NMR spectrum (Fig. 1) features two singlets at 1.23 and 1.40 ppm, each integrating into 18 protons, corresponding to the four *tert*-butyl groups. A peak at 3.33 ppm, integrating to four protons, is associated with the H atoms of the alkyl group. Aromatic signals were observed at 7.05 (H3 and H15) and 6.88 ppm (H1 and H12), with a total integration of four protons. Additionally, a peak at 1.58 ppm indicates the presence of a small amount of water in the product (Sivanesan *et al.*, 2017).

### 3.2. Crystal structure of $[Co(L)_2]ClO_4$ (I)

$[Co(L)_2]ClO_4$ (**I**) (Fig. 2) crystallizes in the monoclinic space group $P2_1/c$. Further crystallographic information can be found in Table 1. The organic ligand $H_4L$ has undergone a chemical reaction and will henceforth be referred to as H*L*. Fig. 2(*a*) illustrates the asymmetric unit, consisting of a cobalt(III) ion, a perchlorate anion and a tridentate 2-[(2-aminoethyl)amino]-4,6-di-*tert*-butylphenolate ligand ($L^-$). Both $Co^{3+}$ and the Cl atom of the perchlorate anion are located at the inversion centres. The asymmetric part of the unit cell contains half of the coordination compound. Therefore, the complete coordination compound is generated by an inversion operation, as shown in Fig. 2(*b*).

The bond lengths of the $L^-$ ligand (see Table S3 in the supporting information) are compared (Fig. 3) with typical bond lengths of other structures containing *o*-aminophenol derivatives like *o*-iminosemiquinonate ($ISQ^{\bullet-}$) and *o*-aminophenolate ($AP^-$) (Nasibipour *et al.*, 2021; Piskunov *et al.*, 2018; Klementyeva *et al.*, 2019; Presow *et al.*, 2011; Brown, 2012; Groom *et al.*, 2016; Broere *et al.*, 2015; Chun *et al.*, 2002). Compared with literature bond lengths, one can assign $L^-$ as a monoanionic non-radical species related to *o*-aminophenolate. This occurs because the ligands do not undergo dehydrogenation to form imide linkages, thus retaining the imine group and classifying them as aminophenolate. When the ligands undergo dehydrogenation and form the amide group, they are classified as iminosemiquinonate and their $ISQ^{\bullet-}$ species is a monoanionic radical (Pinnataip & Lee, 2021; Brown, 2012; Chun *et al.*, 2002; Lever, 2010).

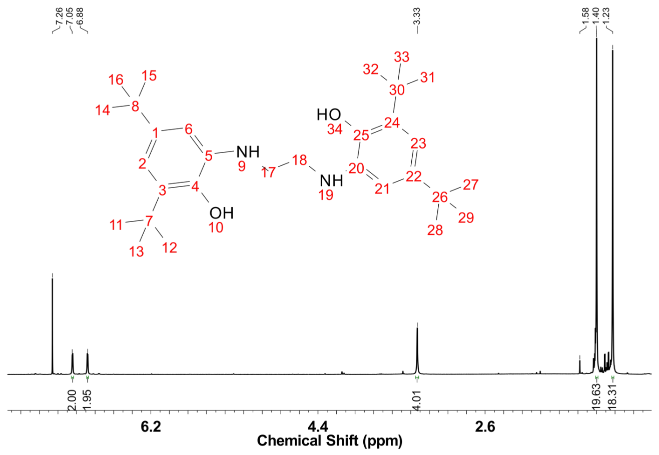

**Figure 1**
$^1$H NMR (400 MHz) spectra of $H_4L$ in $CDCl_3$ (7.26 ppm) at room temperature.

**Table 2**
Selected geometric parameters (Å, °).

| | | | |
|---|---|---|---|
| Co1—O2 | 1.888 (2) | Co1—N12 | 1.949 (3) |
| Co1—N9 | 1.958 (3) | | |
| O2—Co1—N9 | 86.86 (10) | O2—Co1—N12 | 90.19 (12) |
| O2—Co1—N9$^{i}$ | 93.15 (10) | O2—Co1—N12$^{i}$ | 89.81 (12) |

Symmetry code: (i) $-x+1, -y+1, -z+1$.

The $L^-$ ligand is present in the coordination compound with a charge of −1 because it is an $AP^-$ species. This is characterized by homogeneous bond lengths in the aromatic rings (C—C) and the C(ring)—NH(amine) bond has a length typical for a single bond, *i.e.* around 1.4 Å. Therefore, the neutrality of the coordination compound is achieved with a total charge for the two ligands of −2 and the presence of a perchlorate counter-ion with a charge of −1, totaling a charge of −3, which is balanced by the $Co^{3+}$ ion. The metal–ligand, Co—O and Co—N bond lengths support the charge assignment, with values of 1.888 (2)–1.958 (3) Å, which are typical for $Co^{3+}$. Table 2 presents selected bond lengths for compound **I** (Allen *et al.*, 1987).

Further validation of the charge on the metal ion can be obtained by considering the valence bond sum, which aligns with the result obtained from *PLATON* (Spek, 2020), giving a value of 3.32. This value is derived by calculating the valences of the bonds based on the observed bond lengths, following the valence sum rule from Pauling's electrostatic valence concept. Such a calculation provides additional confirmation for the charge assignments by comparing bond-length values with the corresponding charges (Brown, 2009; Pauling, 1929; Altermatt & Brown, 1985).

The metallic centre exhibits a near-octahedral geometry. The values of the $L—M—L$ bond angles range from 86.85 (10) to 93.15 (10)° (Table 2). To analyze this distortion, one approach is to employ Continuous Shape Symmetry Measures (CShMs) software (Alon *et al.*, 2023; Pinsky & Avnir, 1998). Table 3 presents the values found for different geometries, with the lowest value being 0.125, which corresponds to octahedral geometry with $O_h$ point-group symmetry (Guo *et al.*, 2017; Pinsky & Avnir, 1998). This low distortion aligns with the characteristic of a low-spin cobalt(III) complex featuring a small Jahn–Teller distortion, as reflected in the close values for the Co—$L$ bond lengths, making it difficult to distinguish between axial and equatorial positions (Jahn & Teller, 1937).

**Table 3**
Coordination distortion calculated for the coordination environment of $[Co(L)_2]ClO_4$ analysed with CShMs.

| Geometry for $ML_6$ | Point group | Polyhedricity |
|---|---|---|
| Hexagon | $D_{6h}$ | 30.887 |
| Trigonal prism | $D_{3h}$ | 16.130 |
| Pentagonal pyramid | $C_{5v}$ | 28.979 |
| Octahedron | $O_h$ | 0.125 |

The $L^-$ ligand coordinates the metallic centre *via* atoms O2, N9 and N12, constituting a tridentate ligand due to the flexibility of the ethyl group which allows the adoption of a conformation where the coordinated N12 and N9 atoms are in equatorial positions, with bond lengths of 1.949 (3) and 1.958 (3) Å, respectively, while the O2 atom occupies an axial position, with a bond length of 1.888 (2) Å. The Jahn–Teller distortion in the compound is minimal, resulting in a slight compression along the axial axis.

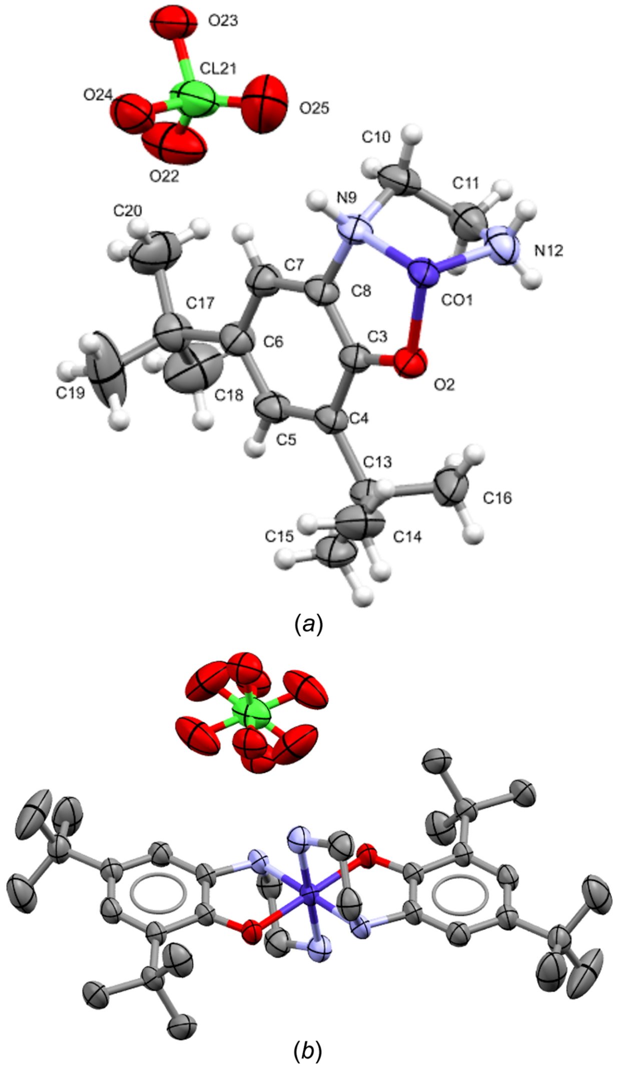

**Figure 2**
Illustration of **I**, with displacement ellipsoids represented with the 50% probability level. (*a*) The asymmetric part of the unit cell. (*b*) The molecular structure of **I**. H atoms have been omitted for clarity.

**Table 4**
Hydrogen-bond geometry (Å, °).

| $D$—H···$A$ | $D$—H | H···$A$ | $D$···$A$ | $D$—H···$A$ |
|---|---|---|---|---|
| N9—H9···O25 | 0.99 | 2.22 | 3.158 (11) | 158 |
| N9—H9···O23$^{ii}$ | 0.99 | 2.09 | 3.038 (7) | 160 |
| N9—H9···O24$^{ii}$ | 0.99 | 2.44 | 3.191 (7) | 132 |
| C7—H7···O22 | 0.94 | 2.51 | 3.263 (8) | 138 |
| C10—H10$A$···O23$^{iii}$ | 0.98 | 2.59 | 3.271 (6) | 127 |
| C11—H11$B$···O22$^{iv}$ | 0.98 | 2.37 | 3.314 (8) | 162 |
| C14—H14$A$···O2 | 0.97 | 2.43 | 3.064 (4) | 123 |
| C16—H16$C$···O2 | 0.97 | 2.38 | 3.018 (5) | 123 |
| N12—H12$A$···O24$^{iv}$ | 0.84 (2) | 2.33 (4) | 3.051 (6) | 144 (5) |
| N12—H12$B$···O23$^{v}$ | 0.82 (2) | 2.78 (4) | 3.473 (6) | 142 (5) |

Symmetry codes: (ii) $-x+1, -y, -z+1$; (iii) $-x+1, y+\frac{1}{2}, -z+\frac{3}{2}$; (iv) $x, -y+\frac{1}{2}, z+\frac{1}{2}$; (v) $x, y+1, z$.

The crystal packing of compound **I** (Fig. 4) shows the perchlorate anion sitting over an inversion centre and the structure was refined with a disordered position (Kratzert *et al.*, 2015). The interactions are due to the $ClO_4^-$ anion localized in the network cavity; in this way, the anion acts as a hydrogen-bond acceptor interacting with the $L^-$ ligand. Each $L^-$ ligand interacts with three $ClO_4^-$ anions; the values of the hydrogen-bond lengths are shown in Table 4. To support our attempt to understand how these interactions operate in the crystal, the Hirshfeld surface was calculated (Fig. 5).

The analysis of the Hirshfeld surface can be supported by a 2D mapping, which quantitatively summarizes the types of intermolecular contacts present. The complete 2D fingerprint for compound **I** [Fig. 6(*a*)] illustrates the occurrence and frequency of all interactions in the packing, as well as the related geometrical parameters. Fig. 6(*b*) focuses on the H···H interactions, which are primally repulsive and account for 79.9% of the surface contribution. Fig. 6(*c*) illustrates the C—H···O interactions, specifically between the H atoms of H$L$ and the perchlorate O atoms. The interactions highlighted

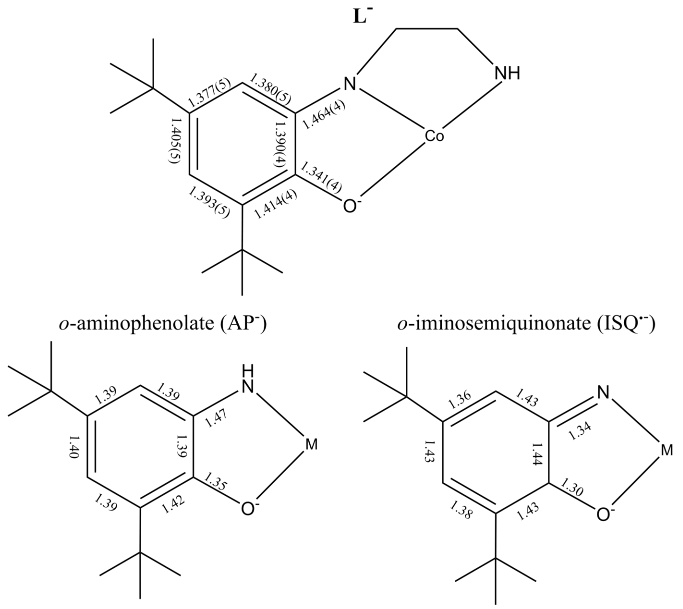

**Figure 3**
The values of the bond lengths for an aromatic ring of $L^-$ and the values typical for the ISQ$^{\bullet-}$ and AP$^-$ species according to Broere *et al.* (2015).

in the red circle correspond to those described in Table 4. These interactions contribute 16.5% to the surface. Fig. 6(*b*) shows the C—H···C interactions, which contribute 3.6% to the surface.

### 4. Conclusion

We report the unpublished structure of a coordination compound of cobalt and a redox-active ligand. Starting from the redox-active ligand 2,4-di-*tert*-butyl-6-({2-[(3,5-di-*tert*-butyl-2-hydroxyphenyl)amino]ethyl}amino)phenol ($H_4L$), it was observed that during the coordination reaction, the ligand has undergone a degradation reaction leading to the formation of 2-[(2-aminoethyl)amino]-4,6-di-*tert*-butylphenol (H$L$). H$L$ then complexes with $Co^{III}$ to form $[Co(L)_2]ClO_4$. We propose a reaction that elucidates the formation of the $L^-$ ligand and the oxidation of the metal ion, based on the reported reactions of catechol ligands. The structure parameter data are discussed and correlated with the Continuous Shape Symmetry Measures approach and Hirshfeld surface analysis.

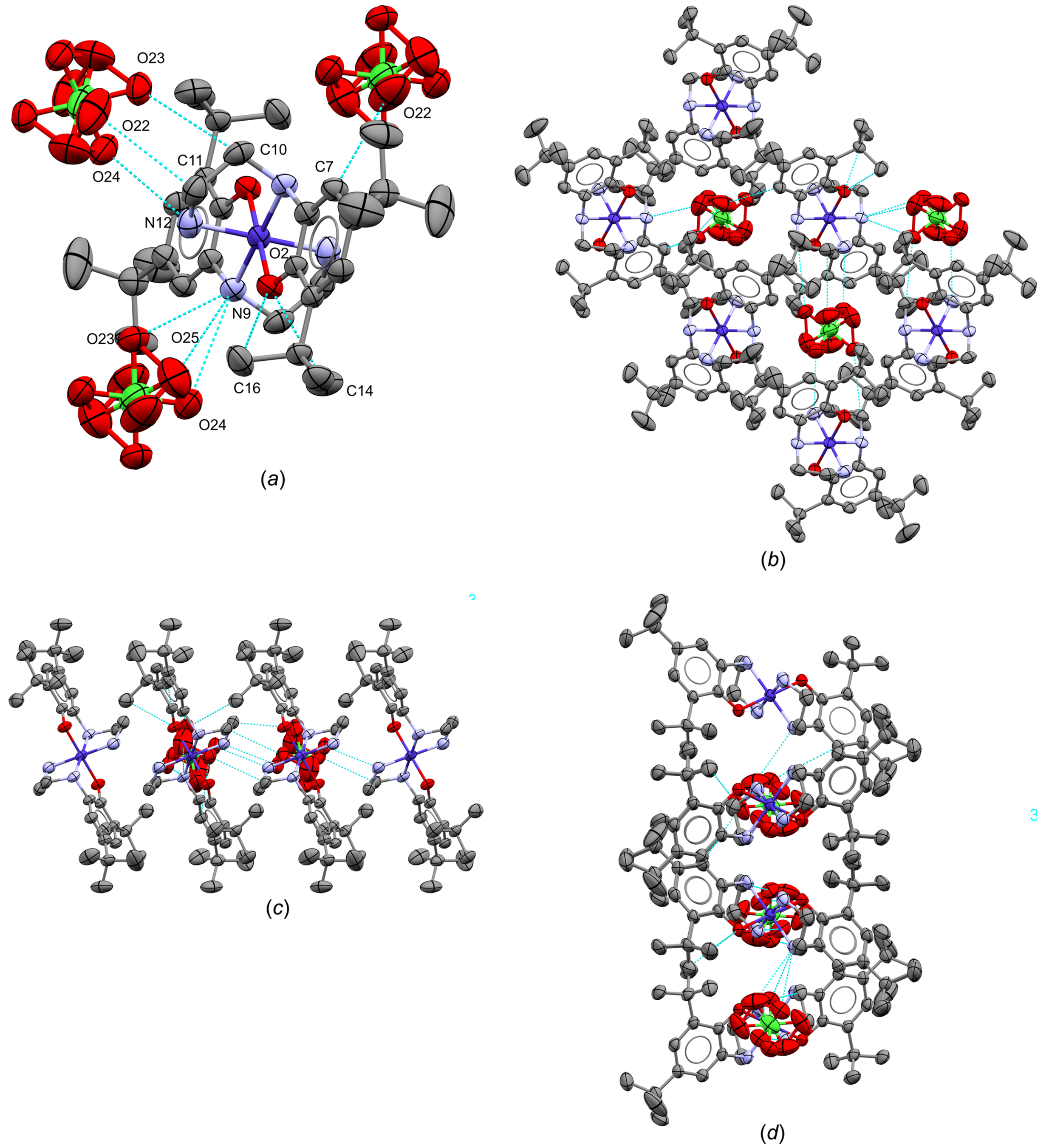

**Figure 4**
Crystal packing diagrams with displacement ellipsoids represented with the 50% probability level. (*a*) The molecular interactions, viewed along (*b*) the *a* axis, (*c*) the *b* axis and (*d*) the *c* axis. H atoms have been omitted for clarity.

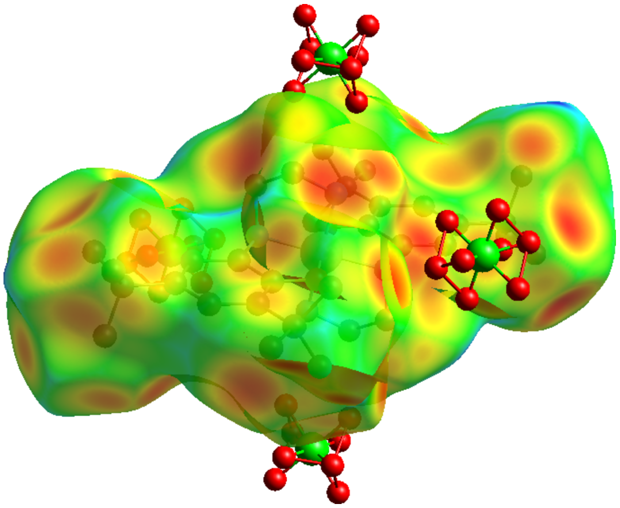

**Figure 5**
A view of the Hirshfeld surface mapped over $d_{norm}$ for **I** with four perchlorates.

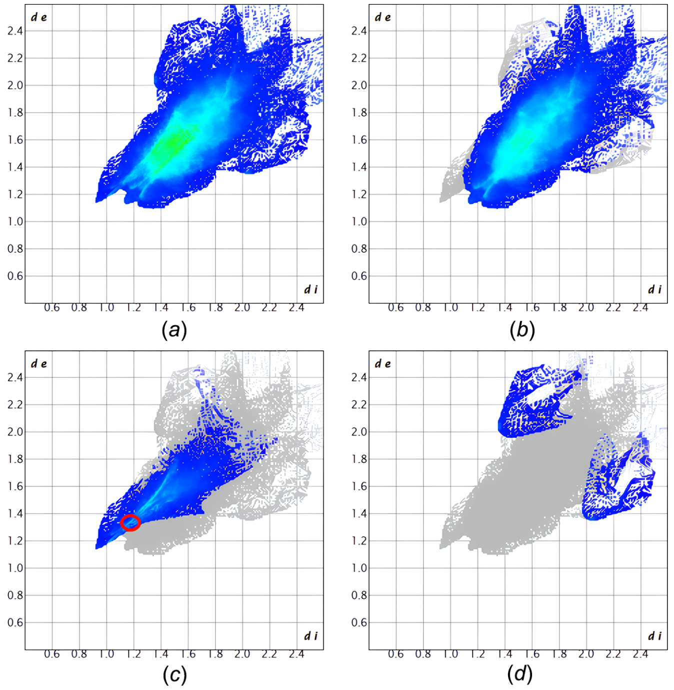

**Figure 6**
Chemical diagram for **I**, showing (*a*) the full 2D fingerprint plot and for (*b*) H···H, (*c*) C—H···O and (*d*) C—H···C contacts.

### Acknowledgements

We are grateful to LabCri at Universidade Federal de Minas Gerais, Brazil, for supporting us in the structural characterization. We also thank CAPES, FAPES and CNPq for their financial support.

### Conflict of interest

All authors hereby confirm that this work is entirely original, has not been published previously and is not currently under consideration for publication elsewhere. Furthermore, the authors declare that there are no conflicts of interest associated with this work.

### Funding information

Funding for this research was provided by: FAPES (award No. 922/2023). The Article Processing Charge for the publication of this research was funded by the Coordenação de Aperfeiçoamento de Pessoal de Nível Superior – Brasil (CAPES) (ROR identifier: 00x0ma614).

### References

Allen, F. H., Kennard, O., Watson, D. G., Brammer, L., Orpen, A. G. & Taylor, R. (1987). *J. Chem. Soc. Perkin Trans. 2*, pp. S1–19.
Alon, G., Ben-Haim, Y. & Tuvi-Arad, I. (2023). *J. Cheminform.* **15**, 106.
Altermatt, D. & Brown, I. D. (1985). *Acta Cryst.* B**41**, 240–244.
Broere, D. L. J., Plessius, R. & van der Vlugt, J. I. (2015). *Chem. Soc. Rev.* **44**, 6886–6915.
Brown, I. D. (2009). *Chem. Rev.* **109**, 6858–6919.
Brown, S. N. (2012). *Inorg. Chem.* **51**, 1251–1260.
Chun, H., Chaudhuri, P., Weyhermüller, T. & Wieghardt, K. (2002). *Inorg. Chem.* **41**, 790–795.
Clark, R. C. & Reid, J. S. (1995). *Acta Cryst.* A**51**, 887–897.
Dolomanov, O. V., Bourhis, L. J., Gildea, R. J., Howard, J. A. K. & Puschmann, H. (2009). *J. Appl. Cryst.* **42**, 339–341.
Gransbury, G. K., Boulon, M., Petrie, S., Gable, R. W., Mulder, R. J., Sorace, L., Stranger, R. & Boskovic, C. (2019). *Inorg. Chem.* **58**, 4230–4243.
Groom, C. R., Bruno, I. J., Lightfoot, M. P. & Ward, S. C. (2016). *Acta Cryst.* B**72**, 171–179.
Guo, X. H., Sun, Y. F., Han, S. Z., Miao, J., Cui, M. & Wang, J. B. (2017). *J. Biol. Regul. Homeost. Agents*, **31**, 389–394.
Hathwar, V. R., Stingaciu, M., Richter, B., Overgaard, J. & Iversen, B. B. (2017). *Acta Cryst.* B**73**, 304–312.
Jahn, H. A. & Teller, E. (1937). *Proc. R. Soc. London*, **161**, 220–235.
Klementyeva, S. V., Lukoyanov, A. N., Afonin, M. Y., Mörtel, M., Smolentsev, A. I., Abramov, P. A., Starikova, A. A., Khusniyarov, M. M. & Konchenko, S. N. (2019). *Dalton Trans.* **48**, 3338–3348.
Kratzert, D., Holstein, J. J. & Krossing, I. (2015). *J. Appl. Cryst.* **48**, 933–938.
Lever, A. B. P. (2010). *Coord. Chem. Rev.* **254**, 1397–1405.
Macrae, C. F., Sovago, I., Cottrell, S. J., Galek, P. T. A., McCabe, P., Pidcock, E., Platings, M., Shields, G. P., Stevens, J. S., Towler, M. & Wood, P. A. (2020). *J. Appl. Cryst.* **53**, 226–235.
Min, K. S., Weyhermüller, T., Bothe, E. & Wieghardt, K. (2004). *Inorg. Chem.* **43**, 2922–2931.
Nasibipour, M., Safaei, E., Moaddeli, A., Masoumpour, M. S. & Wojtczak, A. (2021). *RSC Adv.* **11**, 12845–12859.
Panja, A., Jana, N. C., Patra, M., Brandão, P., Moore, C. E., Eichhorn, D. M. & Frontera, A. (2016). *J. Mol. Catal. A Chem.* **412**, 56–66.
Pauling, L. (1929). *J. Am. Chem. Soc.* **51**, 1010–1026.
Pinnataip, R. & Lee, B. P. (2021). *ACS Omega* **6**, 5113–5118.
Pinsky, M. & Avnir, D. (1998). *Inorg. Chem.* **37**, 5575–5582.
Piskunov, A. V., Pashanova, K. I., Bogomyakov, A. S., Smolyaninov, I. V., Starikov, A. G. & Fukin, G. K. (2018). *Dalton Trans.* **47**, 15049–15060.
Pour, Y. S., Safaei, E., Wojtczak, A. & Jagličić, Z. (2020). *Polyhedron*, **187**, 114620.
Presow, S. R., Ghosh, M., Bill, E., Weyhermüller, T. & Wieghardt, K. (2011). *Inorg. Chim. Acta*, **374**, 226–239.

Rigaku OD (2022). *CrysAlis PRO*. Rigaku Oxford Diffraction Ltd, Yarnton, Oxfordshire, England.
Sheldrick, G. M. (2015*a*). *Acta Cryst.* A**71**, 3–8.
Sheldrick, G. M. (2015*b*). *Acta Cryst.* C**71**, 3–8.
Shultz, D. A. (2003). *Valence Tautomerism in Dioxolene Complexes of Cobalt*, in *Molecules to Materials*, Part 2, edited by J. S. Miller & M. Drillon, pp. 281–306. New York: John Wiley & Sons Inc.
Sivanesan, D., Youn, M. H., Park, K. T., Kim, H. J. & Jeong, S. K. (2017). *Cryst. Growth Des.* **17**, 4504–4510.
Spackman, M. A. & Jayatilaka, D. (2009). *CrystEngComm*, **11**, 19–32.
Spackman, P. R., Turner, M. J., McKinnon, J. J., Wolff, S. K., Grimwood, D. J., Jayatilaka, D. & Spackman, M. A. (2021). *J. Appl. Cryst.* **54**, 1006–1011.
Spek, A. L. (2020). *Acta Cryst.* E**76**, 1–11.
Tezgerevska, T., Rousset, E., Gable, R. W., Jameson, G. N. L., Sañudo, E. C., Starikova, A. & Boskovic, C. (2019). *Dalton Trans.* **48**, 11674–11689.

### supporting information

*Acta Cryst.* (2025). C**81**, 358-364 [https://doi.org/10.1107/S2053229625004309]

### Synthesis and structure analysis of a cobalt(III) coordination compound obtained from a redox-active phenolate ligand and cobalt(II)

**Carlos Eduardo Tartaglia Bruzeguini, Rodrigo Iraha Bezerra and Marcos Antonio Ribeiro**

### Computing details

### Bis{2-[(2-aminoethyl)amino]-4,6-di-*tert*-butylphenolato-$\kappa^3N,N',O$}cobalt(III) perchlorate

*Crystal data*

$[Co(C_{16}H_{27}N_2O)_2]ClO_4$
$M_r = 685.17$
Monoclinic, $P2_1/c$
$a = 15.4774$ (10) Å
$b = 10.4931$ (4) Å
$c = 11.5251$ (5) Å
$\beta = 108.170$ (6)°
$V = 1778.41$ (16) Å$^3$
$Z = 2$
$F(000) = 732$
$D_x = 1.280$ Mg m$^{-3}$
Cu $K\alpha$ radiation, $\lambda = 1.54184$ Å
Cell parameters from 5181 reflections
$\theta = 3.0$–76.6°
$\mu = 4.84$ mm$^{-1}$
$T = 250$ K
Block, yellow
0.09 × 0.08 × 0.03 mm

*Data collection*

Rigaku XtaLAB Synergy Dualflex diffractometer with a HyPix detector
Radiation source: micro-focus sealed X-ray tube, PhotonJet (Cu) X-ray Source
Mirror monochromator
Detector resolution: 10.0000 pixels mm$^{-1}$
$\omega$ scans
Absorption correction: analytical [CrysAlis PRO (Rigaku OD, 2022) based on expressions derived by Clark & Reid (1995)]
$T_{min} = 0.953$, $T_{max} = 0.983$
18781 measured reflections
3366 independent reflections
2856 reflections with $I > 2\sigma(I)$
$R_{int} = 0.076$
$\theta_{max} = 70.0°$, $\theta_{min} = 3.0°$
$h = -18 \rightarrow 18$
$k = -12 \rightarrow 12$
$l = -14 \rightarrow 11$

*Refinement*

Refinement on $F^2$
Least-squares matrix: full
$R[F^2 > 2\sigma(F^2)] = 0.067$
$wR(F^2) = 0.173$
$S = 1.11$
3366 reflections
234 parameters
77 restraints
Primary atom site location: dual
Hydrogen site location: mixed
H atoms treated by a mixture of independent and constrained refinement
$w = 1/[\sigma^2(F_o^2) + (0.0816P)^2 + 1.1682P]$ where $P = (F_o^2 + 2F_c^2)/3$
$(\Delta/\sigma)_{max} < 0.001$
$\Delta\rho_{max} = 0.62$ e Å$^{-3}$
$\Delta\rho_{min} = -0.47$ e Å$^{-3}$

*Special details*

**Geometry**. All esds (except the esd in the dihedral angle between two l.s. planes) are estimated using the full covariance matrix. The cell esds are taken into account individually in the estimation of esds in distances, angles and torsion angles; correlations between esds in cell parameters are only used when they are defined by crystal symmetry. An approximate (isotropic) treatment of cell esds is used for estimating esds involving l.s. planes.

**Refinement**. Single-crystal X-ray diffraction measurements were performed on an Oxford Diffraction Synergy diffractometer. Measurements were performed using the radiation source Mo *Kα* (*λ* = 0,7107 Å) and the temperature control device CryojetXL was used. Data integration and scaling of reflection intensities were performed in the *CrysAlis PRO* (Oxford Diffraction, 2022). The structures were solved and refined using the Dual-Space method in the program *SHELXT2018* (Sheldrick, 2015*a*) and refinement by *SHELXL2018* (Sheldrick, 2015*b*) using the least-squares method. The *OLEX2* (Dolomanov *et al.*, 2009) package was used to support the solution and refinement. Illustrations presented were realized in the software *Mercury* (Macrae *et al.*, 2020) and rendered using the *PovRay* tool. The Hirshfeld surfaces were calculated using *CrystalExplorer* (Version 21.5; Spackman & Jayatilaka, 2009; Spackman *et al.*, 2021).

*Fractional atomic coordinates and isotropic or equivalent isotropic displacement parameters (Å$^2$)*

| | $x$ | $y$ | $z$ | $U_{iso}$*/$U_{eq}$ | Occ. (<1) |
|---|---|---|---|---|---|
| Co1 | 0.500000 | 0.500000 | 0.500000 | 0.0380 (2) | |
| O2 | 0.39751 (15) | 0.5688 (2) | 0.38091 (19) | 0.0433 (5) | |
| N9 | 0.42438 (19) | 0.3510 (3) | 0.5024 (2) | 0.0425 (6) | |
| H9 | 0.457055 | 0.271675 | 0.495582 | 0.051* | |
| N12 | 0.4576 (2) | 0.5704 (3) | 0.6291 (3) | 0.0525 (8) | |
| C8 | 0.3419 (2) | 0.3639 (3) | 0.3977 (3) | 0.0402 (7) | |
| C3 | 0.3309 (2) | 0.4825 (3) | 0.3411 (3) | 0.0376 (7) | |
| C4 | 0.2495 (2) | 0.5076 (3) | 0.2458 (3) | 0.0384 (7) | |
| C7 | 0.2782 (2) | 0.2679 (3) | 0.3622 (3) | 0.0448 (8) | |
| H7 | 0.288719 | 0.189085 | 0.402893 | 0.054* | |
| C6 | 0.1992 (2) | 0.2871 (3) | 0.2673 (3) | 0.0441 (8) | |
| C5 | 0.1874 (2) | 0.4078 (3) | 0.2119 (3) | 0.0435 (8) | |
| H5 | 0.133708 | 0.422266 | 0.147141 | 0.052* | |
| C13 | 0.2311 (2) | 0.6381 (3) | 0.1842 (3) | 0.0447 (8) | |
| C17 | 0.1257 (3) | 0.1844 (4) | 0.2217 (3) | 0.0536 (9) | |
| C10 | 0.4045 (3) | 0.3544 (4) | 0.6214 (3) | 0.0543 (9) | |
| H10A | 0.457809 | 0.326620 | 0.688096 | 0.065* | |
| H10B | 0.353481 | 0.297852 | 0.618541 | 0.065* | |
| C11 | 0.3811 (3) | 0.4898 (4) | 0.6407 (4) | 0.0574 (10) | |
| H11A | 0.323882 | 0.514417 | 0.579314 | 0.069* | |
| H11B | 0.374232 | 0.499994 | 0.721947 | 0.069* | |
| C15 | 0.1373 (3) | 0.6465 (4) | 0.0882 (3) | 0.0602 (10) | |
| H15A | 0.133690 | 0.585936 | 0.023312 | 0.090* | |
| H15B | 0.127771 | 0.732009 | 0.054677 | 0.090* | |
| H15C | 0.090833 | 0.626857 | 0.125827 | 0.090* | |
| C14 | 0.3028 (3) | 0.6657 (4) | 0.1215 (4) | 0.0669 (11) | |
| H14A | 0.362334 | 0.670069 | 0.182383 | 0.100* | |
| H14B | 0.289203 | 0.746387 | 0.078584 | 0.100* | |
| H14C | 0.302205 | 0.598187 | 0.063820 | 0.100* | |
| C16 | 0.2345 (3) | 0.7402 (4) | 0.2813 (4) | 0.0685 (12) | |
| H16A | 0.192129 | 0.718033 | 0.324764 | 0.103* | |
| H16B | 0.218054 | 0.822261 | 0.241865 | 0.103* | |

| | | | | | |
|---|---|---|---|---|---|
| H16C | 0.295581 | 0.744674 | 0.338473 | 0.103* | |
| C20 | 0.1392 (4) | 0.0747 (5) | 0.3116 (5) | 0.0926 (17) | |
| H20A | 0.195361 | 0.030663 | 0.316858 | 0.139* | |
| H20B | 0.088566 | 0.015883 | 0.283952 | 0.139* | |
| H20C | 0.142199 | 0.107631 | 0.391434 | 0.139* | |
| C18 | 0.0318 (3) | 0.2405 (6) | 0.2113 (6) | 0.0932 (17) | |
| H18A | 0.032106 | 0.272980 | 0.290190 | 0.140* | |
| H18B | −0.014075 | 0.174564 | 0.184893 | 0.140* | |
| H18C | 0.018155 | 0.309355 | 0.152150 | 0.140* | |
| C19 | 0.1262 (5) | 0.1364 (7) | 0.0983 (5) | 0.126 (3) | |
| H19A | 0.121169 | 0.208008 | 0.043314 | 0.189* | |
| H19B | 0.075214 | 0.079194 | 0.065260 | 0.189* | |
| H19C | 0.182557 | 0.091207 | 0.107038 | 0.189* | |
| H12A | 0.501 (3) | 0.569 (5) | 0.695 (3) | 0.091 (17)* | |
| Cl21 | 0.500000 | 0.000000 | 0.500000 | 0.0787 (5) | |
| O25 | 0.5480 (7) | 0.1050 (7) | 0.5577 (8) | 0.126 (3) | 0.5 |
| O23 | 0.5025 (4) | −0.1124 (5) | 0.5836 (5) | 0.0691 (16) | 0.5 |
| O24 | 0.5342 (5) | −0.0636 (6) | 0.4071 (5) | 0.0726 (16) | 0.5 |
| O22 | 0.4006 (4) | 0.0097 (9) | 0.4354 (7) | 0.108 (3) | 0.5 |
| H12B | 0.441 (4) | 0.645 (2) | 0.617 (5) | 0.10 (2)* | |

*Atomic displacement parameters (Å$^2$)*

| | $U^{11}$ | $U^{22}$ | $U^{33}$ | $U^{12}$ | $U^{13}$ | $U^{23}$ |
|---|---|---|---|---|---|---|
| Co1 | 0.0314 (4) | 0.0388 (4) | 0.0350 (4) | 0.0033 (3) | −0.0023 (3) | −0.0024 (3) |
| O2 | 0.0348 (13) | 0.0392 (12) | 0.0450 (12) | 0.0001 (10) | −0.0035 (10) | 0.0006 (9) |
| N9 | 0.0346 (15) | 0.0440 (14) | 0.0389 (13) | 0.0064 (12) | −0.0032 (11) | 0.0027 (11) |
| N12 | 0.0452 (19) | 0.060 (2) | 0.0452 (16) | 0.0038 (17) | 0.0035 (14) | −0.0091 (15) |
| C8 | 0.0299 (17) | 0.0438 (16) | 0.0388 (15) | 0.0048 (14) | −0.0009 (13) | 0.0004 (13) |
| C3 | 0.0339 (18) | 0.0375 (16) | 0.0357 (15) | 0.0047 (13) | 0.0025 (13) | 0.0003 (12) |
| C4 | 0.0343 (18) | 0.0416 (16) | 0.0331 (15) | 0.0034 (14) | 0.0017 (13) | 0.0001 (12) |
| C7 | 0.041 (2) | 0.0415 (17) | 0.0458 (17) | 0.0018 (15) | 0.0053 (15) | 0.0039 (13) |
| C6 | 0.0346 (19) | 0.0450 (17) | 0.0473 (17) | −0.0004 (15) | 0.0050 (14) | 0.0001 (14) |
| C5 | 0.0337 (18) | 0.0509 (18) | 0.0387 (16) | 0.0051 (15) | 0.0008 (13) | 0.0030 (13) |
| C13 | 0.045 (2) | 0.0429 (17) | 0.0390 (16) | 0.0077 (15) | 0.0019 (14) | 0.0052 (13) |
| C17 | 0.041 (2) | 0.056 (2) | 0.057 (2) | −0.0094 (17) | 0.0058 (16) | 0.0004 (17) |
| C10 | 0.051 (2) | 0.067 (2) | 0.0374 (17) | −0.0012 (19) | 0.0026 (16) | 0.0117 (16) |
| C11 | 0.055 (2) | 0.071 (3) | 0.0423 (19) | 0.005 (2) | 0.0097 (17) | −0.0038 (16) |
| C15 | 0.054 (2) | 0.054 (2) | 0.057 (2) | 0.0122 (19) | −0.0052 (18) | 0.0084 (17) |
| C14 | 0.061 (3) | 0.071 (3) | 0.065 (2) | 0.001 (2) | 0.014 (2) | 0.024 (2) |
| C16 | 0.074 (3) | 0.048 (2) | 0.066 (2) | 0.014 (2) | −0.003 (2) | −0.0110 (18) |
| C20 | 0.085 (4) | 0.063 (3) | 0.107 (4) | −0.025 (3) | −0.003 (3) | 0.021 (3) |
| C18 | 0.044 (3) | 0.090 (4) | 0.133 (5) | −0.008 (2) | 0.010 (3) | 0.022 (3) |
| C19 | 0.138 (6) | 0.149 (6) | 0.108 (4) | −0.092 (5) | 0.062 (4) | −0.076 (4) |
| Cl21 | 0.0983 (13) | 0.0877 (11) | 0.0496 (8) | 0.0300 (10) | 0.0225 (8) | 0.0144 (7) |
| O25 | 0.176 (9) | 0.085 (5) | 0.110 (6) | −0.013 (6) | 0.034 (6) | −0.020 (4) |
| O23 | 0.082 (4) | 0.068 (3) | 0.053 (3) | 0.016 (3) | 0.016 (3) | 0.016 (3) |
| O24 | 0.089 (5) | 0.079 (4) | 0.051 (3) | 0.016 (4) | 0.023 (3) | 0.004 (3) |

| | | | | | | |
|---|---|---|---|---|---|---|
| O22 | 0.102 (6) | 0.148 (8) | 0.072 (4) | 0.055 (4) | 0.025 (4) | 0.030 (4) |

*Geometric parameters (Å, º)*

| | | | |
|---|---|---|---|
| Co1—O2 | 1.888 (2) | C10—H10A | 0.9800 |
| Co1—O2$^{i}$ | 1.888 (2) | C10—H10B | 0.9800 |
| Co1—N9$^{i}$ | 1.958 (3) | C10—C11 | 1.500 (6) |
| Co1—N9 | 1.958 (3) | C11—H11A | 0.9800 |
| Co1—N12$^{i}$ | 1.949 (3) | C11—H11B | 0.9800 |
| Co1—N12 | 1.949 (3) | C15—H15A | 0.9700 |
| O2—C3 | 1.341 (4) | C15—H15B | 0.9700 |
| N9—H9 | 0.9900 | C15—H15C | 0.9700 |
| N9—C8 | 1.464 (4) | C14—H14A | 0.9700 |
| N9—C10 | 1.497 (4) | C14—H14B | 0.9700 |
| N12—C11 | 1.495 (6) | C14—H14C | 0.9700 |
| N12—H12A | 0.84 (2) | C16—H16A | 0.9700 |
| N12—H12B | 0.823 (18) | C16—H16B | 0.9700 |
| C8—C3 | 1.390 (4) | C16—H16C | 0.9700 |
| C8—C7 | 1.380 (5) | C20—H20A | 0.9700 |
| C3—C4 | 1.414 (4) | C20—H20B | 0.9700 |
| C4—C5 | 1.393 (5) | C20—H20C | 0.9700 |
| C4—C13 | 1.527 (4) | C18—H18A | 0.9700 |
| C7—H7 | 0.9400 | C18—H18B | 0.9700 |
| C7—C6 | 1.377 (5) | C18—H18C | 0.9700 |
| C6—C5 | 1.405 (5) | C19—H19A | 0.9700 |
| C6—C17 | 1.536 (5) | C19—H19B | 0.9700 |
| C5—H5 | 0.9400 | C19—H19C | 0.9700 |
| C13—C15 | 1.529 (5) | Cl21—O25 | 1.379 (6) |
| C13—C14 | 1.528 (6) | Cl21—O23 | 1.516 (5) |
| C13—C16 | 1.538 (5) | Cl21—O23$^{ii}$ | 1.516 (5) |
| C17—C20 | 1.519 (6) | Cl21—O24 | 1.491 (5) |
| C17—C18 | 1.538 (7) | Cl21—O24$^{ii}$ | 1.491 (5) |
| C17—C19 | 1.511 (6) | Cl21—O22 | 1.489 (6) |
| | | | |
| O2—Co1—O2$^{i}$ | 180.0 | H10A—C10—H10B | 108.6 |
| O2—Co1—N9 | 86.86 (10) | C11—C10—H10A | 110.4 |
| O2$^{i}$—Co1—N9 | 93.14 (10) | C11—C10—H10B | 110.4 |
| O2—Co1—N9$^{i}$ | 93.15 (10) | N12—C11—C10 | 106.7 (3) |
| O2$^{i}$—Co1—N9$^{i}$ | 86.85 (10) | N12—C11—H11A | 110.4 |
| O2—Co1—N12 | 90.19 (12) | N12—C11—H11B | 110.4 |
| O2—Co1—N12$^{i}$ | 89.81 (12) | C10—C11—H11A | 110.4 |
| O2$^{i}$—Co1—N12 | 89.81 (12) | C10—C11—H11B | 110.4 |
| O2$^{i}$—Co1—N12$^{i}$ | 90.19 (12) | H11A—C11—H11B | 108.6 |
| N9$^{i}$—Co1—N9 | 180.0 | C13—C15—H15A | 109.5 |
| N12—Co1—N9 | 86.95 (13) | C13—C15—H15B | 109.5 |
| N12—Co1—N9$^{i}$ | 93.05 (13) | C13—C15—H15C | 109.5 |
| N12$^{i}$—Co1—N9$^{i}$ | 86.95 (13) | H15A—C15—H15B | 109.5 |
| N12$^{i}$—Co1—N9 | 93.05 (13) | H15A—C15—H15C | 109.5 |

| | |
|---|---|
| N12—Co1—N12$^{i}$ | 180.0 |
| C3—O2—Co1 | 111.85 (18) |
| Co1—N9—H9 | 110.4 |
| C8—N9—Co1 | 106.6 (2) |
| C8—N9—H9 | 110.4 |
| C8—N9—C10 | 112.2 (3) |
| C10—N9—Co1 | 106.8 (2) |
| C10—N9—H9 | 110.4 |
| Co1—N12—H12A | 109 (4) |
| Co1—N12—H12B | 113 (4) |
| C11—N12—Co1 | 108.6 (2) |
| C11—N12—H12A | 109 (4) |
| C11—N12—H12B | 110 (4) |
| H12A—N12—H12B | 107 (5) |
| C3—C8—N9 | 114.7 (3) |
| C7—C8—N9 | 122.4 (3) |
| C7—C8—C3 | 122.9 (3) |
| O2—C3—C8 | 118.4 (3) |
| O2—C3—C4 | 122.9 (3) |
| C8—C3—C4 | 118.7 (3) |
| C3—C4—C13 | 121.3 (3) |
| C5—C4—C3 | 116.6 (3) |
| C5—C4—C13 | 122.1 (3) |
| C8—C7—H7 | 119.9 |
| C6—C7—C8 | 120.3 (3) |
| C6—C7—H7 | 119.9 |
| C7—C6—C5 | 116.8 (3) |
| C7—C6—C17 | 123.3 (3) |
| C5—C6—C17 | 120.0 (3) |
| C4—C5—C6 | 124.8 (3) |
| C4—C5—H5 | 117.6 |
| C6—C5—H5 | 117.6 |
| C4—C13—C15 | 112.7 (3) |
| C4—C13—C14 | 109.3 (3) |
| C4—C13—C16 | 109.1 (3) |
| C15—C13—C16 | 107.2 (3) |
| C14—C13—C15 | 108.2 (3) |
| C14—C13—C16 | 110.3 (4) |
| C6—C17—C18 | 109.8 (3) |
| C20—C17—C6 | 111.6 (3) |
| C20—C17—C18 | 105.6 (4) |
| C19—C17—C6 | 109.6 (3) |
| C19—C17—C20 | 110.5 (5) |
| C19—C17—C18 | 109.7 (5) |
| N9—C10—H10A | 110.4 |
| N9—C10—H10B | 110.4 |
| N9—C10—C11 | 106.6 (3) |
| H15B—C15—H15C | 109.5 |
| C13—C14—H14A | 109.5 |
| C13—C14—H14B | 109.5 |
| C13—C14—H14C | 109.5 |
| H14A—C14—H14B | 109.5 |
| H14A—C14—H14C | 109.5 |
| H14B—C14—H14C | 109.5 |
| C13—C16—H16A | 109.5 |
| C13—C16—H16B | 109.5 |
| C13—C16—H16C | 109.5 |
| H16A—C16—H16B | 109.5 |
| H16A—C16—H16C | 109.5 |
| H16B—C16—H16C | 109.5 |
| C17—C20—H20A | 109.5 |
| C17—C20—H20B | 109.5 |
| C17—C20—H20C | 109.5 |
| H20A—C20—H20B | 109.5 |
| H20A—C20—H20C | 109.5 |
| H20B—C20—H20C | 109.5 |
| C17—C18—H18A | 109.5 |
| C17—C18—H18B | 109.5 |
| C17—C18—H18C | 109.5 |
| H18A—C18—H18B | 109.5 |
| H18A—C18—H18C | 109.5 |
| H18B—C18—H18C | 109.5 |
| C17—C19—H19A | 109.5 |
| C17—C19—H19B | 109.5 |
| C17—C19—H19C | 109.5 |
| H19A—C19—H19B | 109.5 |
| H19A—C19—H19C | 109.5 |
| H19B—C19—H19C | 109.5 |
| O25—Cl21—O23 | 114.4 (5) |
| O25—Cl21—O23$^{ii}$ | 65.6 (5) |
| O25—Cl21—O24$^{ii}$ | 63.6 (5) |
| O25—Cl21—O24 | 116.4 (5) |
| O25—Cl21—O22 | 120.3 (5) |
| O23—Cl21—O23$^{ii}$ | 180.0 |
| O24$^{ii}$—Cl21—O23 | 80.6 (3) |
| O24—Cl21—O23$^{ii}$ | 80.6 (3) |
| O24$^{ii}$—Cl21—O23$^{ii}$ | 99.4 (3) |
| O24—Cl21—O23 | 99.4 (3) |
| O24$^{ii}$—Cl21—O24 | 180.0 (2) |
| O22—Cl21—O23$^{ii}$ | 79.1 (4) |
| O22—Cl21—O23 | 100.9 (4) |
| O22—Cl21—O24$^{ii}$ | 77.6 (4) |
| O22—Cl21—O24 | 102.4 (4) |

| | | | |
|---|---|---|---|
| Co1—O2—C3—C8 | 5.7 (4) | C3—C8—C7—C6 | −0.4 (5) |
| Co1—O2—C3—C4 | −175.5 (2) | C3—C4—C5—C6 | 1.7 (5) |
| Co1—N9—C8—C3 | −11.3 (3) | C3—C4—C13—C15 | −177.1 (3) |
| Co1—N9—C8—C7 | 171.8 (3) | C3—C4—C13—C14 | 62.6 (4) |
| Co1—N9—C10—C11 | 43.7 (3) | C3—C4—C13—C16 | −58.1 (4) |
| Co1—N12—C11—C10 | 37.7 (3) | C7—C8—C3—O2 | −178.9 (3) |
| O2—C3—C4—C5 | 178.4 (3) | C7—C8—C3—C4 | 2.3 (5) |
| O2—C3—C4—C13 | −2.0 (5) | C7—C6—C5—C4 | 0.2 (5) |
| N9—Co1—O2—C3 | −9.9 (2) | C7—C6—C17—C20 | 14.2 (6) |
| $N9^{i}$—Co1—O2—C3 | 170.1 (2) | C7—C6—C17—C18 | 131.0 (4) |
| N9—C8—C3—O2 | 4.2 (4) | C7—C6—C17—C19 | −108.5 (5) |
| N9—C8—C3—C4 | −174.6 (3) | C5—C4—C13—C15 | 2.5 (5) |
| N9—C8—C7—C6 | 176.3 (3) | C5—C4—C13—C14 | −117.8 (4) |
| N9—C10—C11—N12 | −53.5 (4) | C5—C4—C13—C16 | 121.5 (4) |
| $N12^{i}$—Co1—O2—C3 | 83.2 (2) | C5—C6—C17—C20 | −165.7 (4) |
| N12—Co1—O2—C3 | −96.8 (2) | C5—C6—C17—C18 | −49.0 (5) |
| C8—N9—C10—C11 | −72.8 (4) | C5—C6—C17—C19 | 71.6 (5) |
| C8—C3—C4—C5 | −2.8 (5) | C13—C4—C5—C6 | −177.9 (3) |
| C8—C3—C4—C13 | 176.8 (3) | C17—C6—C5—C4 | −179.9 (3) |
| C8—C7—C6—C5 | −0.9 (5) | C10—N9—C8—C3 | 105.3 (3) |
| C8—C7—C6—C17 | 179.2 (3) | C10—N9—C8—C7 | −71.7 (4) |

Symmetry codes: (i) −*x*+1, −*y*+1, −*z*+1; (ii) −*x*+1, −*y*, −*z*+1.

*Hydrogen-bond geometry (Å, º)*

| *D*—H···*A* | *D*—H | H···*A* | *D*···*A* | *D*—H···*A* |
|---|---|---|---|---|
| N9—H9···O25 | 0.99 | 2.22 | 3.158 (11) | 158 |
| N9—H9···$O23^{ii}$ | 0.99 | 2.09 | 3.038 (7) | 160 |
| N9—H9···$O24^{ii}$ | 0.99 | 2.44 | 3.191 (7) | 132 |
| C7—H7···O22 | 0.94 | 2.51 | 3.263 (8) | 138 |
| C10—H10*A*···$O23^{iii}$ | 0.98 | 2.59 | 3.271 (6) | 127 |
| C11—H11*B*···$O22^{iv}$ | 0.98 | 2.37 | 3.314 (8) | 162 |
| C14—H14*A*···O2 | 0.97 | 2.43 | 3.064 (4) | 123 |
| C16—H16*C*···O2 | 0.97 | 2.38 | 3.018 (5) | 123 |
| N12—H12*A*···$O24^{iv}$ | 0.84 (2) | 2.33 (4) | 3.051 (6) | 144 (5) |
| N12—H12*B*···$O23^{v}$ | 0.82 (2) | 2.78 (4) | 3.473 (6) | 142 (5) |

Symmetry codes: (ii) −*x*+1, −*y*, −*z*+1; (iii) −*x*+1, *y*+1/2, −*z*+3/2; (iv) *x*, −*y*+1/2, *z*+1/2; (v) *x*, *y*+1, *z*.
